# Peristaltic Pump–Assisted Fat Harvesting and Closed-Circuit Grafting: Technique and Immediate Cell Membrane Integrity in a Case Series

**DOI:** 10.1093/asjof/ojag122

**Published:** 2026-06-23

**Authors:** Christopher Nichols, Daniel Albershardt

## Abstract

Autologous fat grafting outcomes are influenced by tissue handling during harvest, processing, and reinjection. Traditional vacuum-based suction systems generate variable subatmospheric pressures and may introduce turbulence and shear forces that can affect adipocyte viability. The aim of this study was to describe a reversible peristaltic pump–assisted technique alternative for fat harvest and grafting and to outline its procedural workflow and mechanistic considerations. This report describes a closed-circuit, peristaltic pump–assisted technique based on the authors’ 5-year clinical experience across more than 160 aesthetic and reconstructive procedures. The technique enables bidirectional flow for controlled aspiration and reinjection without direct handling of the tissue. The technique was applied across multiple anatomic regions, including the face, breast, and body. Tumescent infiltration, aspirate, and graft volumes were adjusted based on anatomic location and procedural goals. Intraoperative handling was characterized by continuous flow, minimal air entrainment, and minimal clogging. The technique was used for both aesthetic and reconstructive operations, including breast procedures. Peristaltic pump–assisted fat harvest and grafting represent a closed-circuit, non–vacuum-based approach to autologous fat transfer and an alternative in fat grafting technique. By combining precise pressure regulation with an adaptable closed-loop architecture, this method may contribute to consistent and reproducible graft quality, reduce tissue trauma, and decrease surgeon fatigue. This descriptive report demonstrates technical feasibility and reproducible intraoperative handling characteristics. Further controlled studies are needed to evaluate clinical outcomes and comparative effectiveness.

**Level of Evidence: 4 (Therapeutic)** 
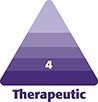

Autologous fat grafting is an integral tool in both reconstructive and aesthetic surgery, where it may contribute to surgical goals, such as volume restoration, contour refinement, soft-tissue augmentation, symmetry improvement, and optimization of tissue-prosthetic interfaces. Success is influenced by tissue handling during harvest, processing, and reinjection. Adipocytes are structurally fragile, and previous studies suggest that excessive subatmospheric pressure, shear forces, and tissue handling conditions during harvest may contribute to adipocyte membrane disruption, increased oil extravasation, and reduction of viable cells essential for long-term graft survival.^1-4^ Despite broad adoption in aesthetic and reconstructive surgery, significant variability persists in the suction pressures, shear profiles, and handling practices employed across operators and devices. These variations may influence tissue handling characteristics and highlight the need for approaches that provide greater procedural consistency and control.

Previous studies evaluating harvest conditions and aspiration techniques have reported variable findings regarding cell preservation, suggesting that pressure magnitude, aspiration mechanics, and tissue handling may influence harvested adipose tissue characteristics. Shiffman and Mirrafati demonstrated that increases in negative pressure during harvest are associated with increased peripheral adipocyte injury, with minimal disruption observed at −250 to 300 mm Hg but increased membrane damage at higher vacuum, such as −700 mm Hg.^5^ Mojallal et al similarly demonstrated that suction conditions influenced stromal vascular fraction (SVF) cell yield, with differences observed across syringe aspiration, traditional suction-assisted liposuction, and power-assisted liposuction (PAL) performed at varying vacuum pressures.^6^ Collectively, these findings suggest that harvest conditions, pressure differential, and tissue handling characteristics may influence adipose tissue properties during harvest.

Fisher et al have further emphasized that the method of harvest itself, not only downstream processing, plays a decisive role in determining graft quality, with higher shear and barotrauma contributing to reduced adipocyte survival and impaired handling characteristics.^7^ These findings reinforce earlier work by Coleman underscoring the importance of atraumatic, low-shear tissue handling throughout the grafting process.^8^ Additional comparative studies evaluating high vs low pressure differential during harvest similarly demonstrate increased adipocyte injury at higher vacuum settings, further supporting the concept of minimizing excessive pressure changes during aspiration.^9^

Peristaltic displacement generates fluid flow through cyclic compression of flexible tubing, creating a controlled movement through the circuit. Because the system is reversible, the same closed circuit may be used for both aspiration and reinjection with minimal manipulation, maintaining sterility and maximizing efficiency without the need for manual open-air exchanges, transfers, or direct handling of the tissue.

This article provides a detailed, systematic description of the peristaltic pump–assisted technique and operative considerations for fat harvest and grafting. The focus is on procedural workflow, technical considerations, and mechanistic principles.

## METHODS

### Peristaltic Pump Systems

Fat harvesting and reinjection were performed using peristaltic pump systems capable of bidirectional flow through sterile tubing in a closed circuit. Early cases were performed using a stand-alone peristaltic pump system (Klein pump, Klein Products, Ontario, CA), followed by later cases utilizing a commercially available reversible peristaltic pump and liposuction platform (AYON Body Contouring System, Apyx Medical, Clearwater, FL). Both systems employ peristaltic displacement to facilitate aspiration and reinjection. Flow reversal was achieved manually in the Klein system and electronically in the Apyx Medical system.

### Bench Testing

Descriptive bench testing was performed to characterize vacuum behavior of the peristaltic pump under controlled conditions. A pressure sensor was positioned along the suction side of the circuit using standard peristaltic pump tubing. Testing was conducted across flow rates of ∼250 to 500 mL/min under both unobstructed flow and simulated obstruction conditions. Obstruction was simulated by partial or complete occlusion of the tubing to approximate cannula blockage.

### Equipment Setup

The peristaltic pump is positioned in-line between the harvest cannula and sterile collection container. Standard commercially available peristaltic pump tubing with an internal diameter of ∼6 mm was utilized (AYON Infiltration Tubing, AYON-IF-TUBE, Apyx Medical). A standard sterile intravenous fluid bag was used for tumescent infiltration and collection. Alternatively, a sterile collection canister could be employed based on surgeon preference. This configuration maintains a continuous closed circuit from the harvest cannula to the collection vessel (Video 1).

### Harvest Technique

Standard tumescent was mixed in a 1 L IV bag (B. Braun Medical Inc., Bethlehem, PA), and infiltration was performed at donor sites using the peristaltic pump before aspiration. This also prewets all the harvesting surfaces within the tubing, preventing tissue contact with dry silicone surfaces. Aspiration pressure was maintained between 100 and 250 mm Hg by adjusting the rotational speed of the occlusion roller. Flow was maintained continuously through adjustment of pump speed. Suction pressure modulation is achieved through variable pump-head speed control without clamping or interrupting suction.

### Processing and Handling

**Figure 1.**
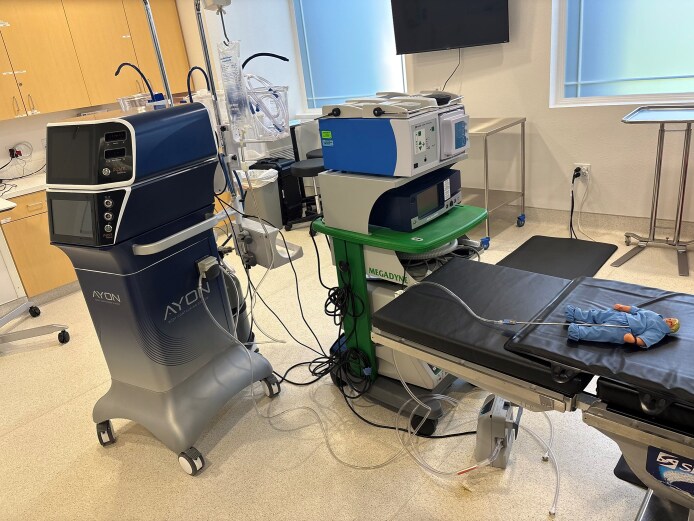
Reversible peristaltic pump in closed-loop configuration showing inline cannula connection and sterile collection bag setup.

Following harvest, the lipoaspirate is allowed to separate by gravity decantation while remaining within the closed-loop collection container (Figure 1). After phase separation, the aqueous infranatant is decanted off using the peristaltic pump itself, without opening the bag or canister.

The decanted fat remains within the original closed environment, ready for reinjection or storage. At this stage, adjuncts (eg, poloxamer 188 [P188], saline washes, or other additives) could be introduced according to surgeon preference.

### Reinjection

The surgeon may then transition from harvest to reinjection because of the reversibility of the pump. Flow direction is reversed, and flow rate reduced to ∼40 mL/min. It is the authors’ preference to connect to a PAL handpiece equipped with an exploded-basket cannula, enabling expansion vibration lipofilling (EVL), as described by Del Vecchio and Wall.^10^ In this configuration, the peristaltic pump provides a controlled fat stream, whereas the oscillatory motion of the PAL handpiece assists with graft delivery.

For small-volume or precise grafting, such as focal contour refinement or grafting of the face or hands, fat is delivered directly through a hand cannula connected to the closed circuit, maintaining a low-pressure environment without the repetitive filling and plunging through Luer-lock connections (1 mm internal diameter) required with manual syringes (Video 2).

### Intraoperative Pearls

1. Optimize flow rate: Maintain aspiration below 300 mm Hg (ideally 100-250 mm Hg) to reduce shear-related adipocyte injury. This typically corresponds to peristaltic pump flow rates of ∼250 to 500 mL/min using ∼6 mm internal diameter tubing. Monitor for cannula obstruction, which may transiently increase vacuum levels.
2. Maintain a continuous fluid column: Ensure tubing is fully primed with liquid to prevent air entrainment, which introduces turbulence and destabilizes laminar flow.
3. Use transparent tubing: Clear tubing permits visualization of flow continuity and early identification of blood in the aspirate, fibrous obstruction, or flow interruption.
4. Aspirate only when needed: Leverage “feathering” the suction with the foot pedal to adjust suction pressure in challenging areas. Keep suction at the minimum needed to move the aspirate through the tubing.
5. Minimize open transfers: The closed-loop system eliminates the need for external syringes or open canisters, potentially reducing contamination risk and mechanical manipulation.
6. Leverage pump reversibility: Transition between harvest and reinjection without breaking the sterile circuit or generating pressure spikes.
7. Select reinjection mode appropriately: Use PAL-assisted EVL for large-volume grafting and direct low-flow peristaltic hand cannula delivery for smaller or delicate regions.
8. Minimize flow restrictions in tubing: Avoid Luer locks, small bore tubing, or small diameter tubing spikes.

Table 1 provides the key parameters for the described peristaltic pump–assisted fat harvest and grafting.

**Table 1.** Recommended Operating Parameters for Peristaltic Pump–Assisted Fat Harvest and Reinjection

Parameter	Recommended range	Rationale
Aspiration pressure	100-250 mm Hg	Minimize barotrauma
Tubing ID	∼6 mm	Prevents flow restriction and Venturi-like shearing forces
Flow rate (harvest)	250-500 mL/min	Efficient, continuous aspiration while maintaining laminar flow
Reinjection rate	∼40 mL/min	Optimized for EVL or hand cannula delivery
Circuit type	Closed-loop sterile tubing	Reduces contamination risk

EVL, expansion vibration lipofilling; ID, inner diameter.

## RESULTS

In bench testing, a vacuum sensor positioned along the suction side of the circuit demonstrated that pump flow rates of ∼250 to 500 mL/min using ∼6 mm internal diameter tubing remained below 250 mm Hg under unobstructed flow conditions, supporting the pressure ranges used clinically. This technique was applied to a range of aesthetic indications and reconstructive breast procedures, across more than 160 fat grafting procedures over 5 years.

Tumescent infiltration volumes, aspirate volumes, and graft volumes were adjusted based on the anatomic region and procedural goals, with larger volumes typically utilized in body and breast applications and smaller volumes in facial and hand procedures. In the author's practice, wet-to-superwet infiltration techniques were generally utilized, with ∼1:1 to 2:1 tumescent infiltration relative to anticipated aspirate volume and a typical dwell time of at least 10 to 15 min before harvest. In the authors’ experience, this approach was associated with a relatively low-blood-content aspirate and concentrated adipose slurry during harvest, although these parameters were not quantitatively measured in the present report. The technique was employed across multiple donor and recipient sites, including the abdomen, flanks, thighs, and back for harvest, and the face, breast, abdomen, hands, and buttocks for grafting.

In the author's experience, harvested adipose demonstrated uniform particulate distribution, and reinjection was characterized by continuous flow with no significant clogging, flow interruption, or pressure fluctuation. Lipoaspirate drawn directly into the collection container demonstrated no visible frothing or air contamination observed, minimal air entrainment, and a minimal oil fraction.

During large-volume grafting, EVL delivered even dispersion (verified with ultrasound on Brazilian Butt Lift [BBL] cases) and favorable handling characteristics, with no palpable nodularity, ridging, or compartmentalization noted on intraoperative or postoperative examination. For smaller-volume applications using the direct hand cannula, controlled peristaltic flow allowed precise deposition with minimal surgeon fatigue based on the author's experience and without the repetitive syringe refilling required with manual injection.

Across all cases in this series, no anecdotal instances of infection, clinically significant fat necrosis, or device-related malfunction were encountered; however, complications were not systematically collected or analyzed in this report.

## DISCUSSION

Peristaltic pump–assisted harvest applies established principles of controlled pressure and fluid dynamics, in which stable laminar flow and low-amplitude pressure variation may preserve delicate cellular structures and minimize mechanical trauma.^11,12^ By avoiding the large subatmospheric-pressure drops, air entrainment, and turbulent flow inherent to traditional vacuum aspiration systems, the technique may potentially reduce much of the destructive shear trauma that has been shown to disrupt adipocyte membranes and compromise graft quality.^5,7,13^ As noted in a comprehensive review by Fontes et al, high negative pressure and shear forces consistently correlate with reduced adipocyte viability and unpredictable graft take.^14^

Peristaltic pumps generate flow through cyclic compression of flexible tubing, resulting in displacement of fluid through the circuit. This mechanism may produce a more continuous flow profile under unobstructed conditions, with different pressure characteristics compared with vacuum-based aspiration systems; however, these effects were not directly measured in the present report. Bench testing demonstrated that unobstructed peristaltic flow is associated with relatively low vacuum levels within the aspiration circuit.

When a peristaltic (displacement) pump is used for aspiration, the vacuum experienced by harvested fat during normal, unobstructed flow is negligible, consisting only of a small, flow-dependent pressure drop required to maintain fluid movement, rather than a sustained system vacuum. Unlike aspiration systems that rely on a sustained vacuum source, a peristaltic pump generates flow through mechanical displacement of fluid within the tubing. Under free-flow conditions (ie, in the absence of cannula occlusion), a minimal localized pressure gradient is generated, sufficient to overcome system resistance and maintain flow without imposing sustained negative pressure on the aspirated material.

In the event of cannula occlusion, a vacuum develops within the tubing on the inlet side of the peristaltic pump. The rate of vacuum buildup is governed by the pump flow rate, the internal volume of the tubing segment between the occlusion and the pump, and the amount of air present in that segment. This vacuum exposure is localized to the segment between the occlusion point and the pump. Fat that has already passed through the pump and been collected downstream is not subjected to this vacuum. Furthermore, the buildup of vacuum is progressive rather than instantaneous, allowing the operator time to recognize the occlusion and stop pump actuation, thereby limiting both the magnitude and duration of exposure.

In contrast, aspiration (vacuum) pump systems maintain a continuous vacuum within the collection canister. This vacuum acts as the driving force for flow and is transmitted throughout the fluid pathway whenever a continuous column of fluid or fat is present. As a result, aspirated fat is continuously exposed to the set vacuum level during both transport through the tubing and residence within the collection canister. Although intermittent air entrainment may transiently reduce vacuum, the system rapidly returns to the set point once fluid continuity is reestablished. Notably, this sustained vacuum exposure occurs during normal operation and does not require occlusion.

These differences highlight a fundamental distinction between the 2 systems: aspiration pumps expose all of the harvested fat to a continuous, system-wide vacuum, whereas peristaltic pumps generate negligible vacuum during normal operation, with vacuum only occurring under occlusion conditions where it is both spatially confined and temporally limited.

The fundamental harvesting and grafting principles remained consistent across systems. The primary difference between platforms is related to the method of flow reversal. With the Klein pump, flow reversal was achieved manually by disengagement of the occlusion rollers and manual reversal of tubing direction. With the AYON system, pump head rotation direction was reversed electronically using an electronic control mechanism. In both configurations, bidirectional peristaltic flow allowed transition from harvest to reinjection within a closed circuit and without exposing the fat in the circuit to open-air desiccation or contaminants. Similar closed-system, peristaltic-assisted fat grafting workflows have been previously described in large-volume gluteal fat grafting procedures, where harvested adipose was processed within a closed circuit and reinjected using peristaltic pump systems.^15^

Integration of PAL with EVL introduces controlled oscillatory motion at the cannula tip, facilitating tissue expansion and uniform fat distribution. Del Vecchio and Wall demonstrated that EVL creates microexpansion channels that distribute graft material efficiently while reducing cannula resistance, thereby enhancing both precision and surgeon ergonomics.^10^ In the author's experience, EVL benefits from a continuous, pressure-stable fat stream, provided by a peristaltic-controlled flow source. Ferretti et al similarly reported that peristaltic pump–driven aspiration reduced peak vacuum forces and mechanical trauma compared with mechanical suction, supporting the hypothesis that pressure-stable, low-shear flow may better preserve adipocyte integrity.^16^

In the authors’ experience, the syringe-free workflow may reduce surgeon fatigue, increase operating room (OR) efficiency, and facilitate more consistent control of cannula-tip position during graft delivery. This ergonomic advantage may support improved consistency in deposition patterns, particularly during large-volume procedures.

In reconstructive breast surgery, fat grafting is used for contour refinement, implant coverage, and correction of soft-tissue deficiencies. Techniques that minimize mechanical trauma during harvest and reinjection may be particularly relevant in these settings, where graft retention and tissue quality are critical considerations. In the author's experience, for small, precise regions such as the face or hand, direct peristaltic-line cannula delivery provides accurate deposition without the shear or variable pressures associated with manual syringe plunging. The authors anticipate further research to refine and miniaturize low-amplitude PAL systems designed specifically for delicate applications, ideally incorporating real-time pressure and flow feedback to further automate graft placement and reduce technique variability.

Beyond mechanical considerations, the biologic mechanisms of fat graft survival further support the rationale for a low-trauma, pressure-stable harvest/grafting method. Pu describes 2 complementary mechanisms of fat graft survival—graft survival and graft replacement—both of which depend on early preservation of viable adipocytes and timely neovascularization.^17^ Excessive mechanical trauma during harvest may reduce the pool of adipocytes capable of early plasmatic imbibition, whereas high-pressure injection may impede microvascular ingrowth.

Similarly, Yoshimura et al has shown that adipose-derived stem/progenitor cells (ASCs) play a central role in postgrafting tissue remodeling, particularly between Days 3 and 7 after grafting, where they contribute to angiogenesis, adipogenesis, and replacement of nonviable adipocytes.^12^ Importantly, aspirated fat has been shown to contain significantly fewer ASCs than intact adipose tissue, a limitation that may be exacerbated by mechanical trauma during harvest. Techniques that minimize shear and pressure variation may therefore help preserve not only adipocytes but particularly the supportive SVF/ASC population, as well as the architectural relationships between these cells, which are crucial for long-term graft survival.

Taken together, the mechanical and biological considerations provide impetus for evaluating different harvesting approaches. This peristaltic pump technique represents a non–vacuum-based approach to fat harvest and grafting. Controlled clinical studies are needed to evaluate clinical outcomes and reproducibility.

Closed-circuit processing approaches may have broader implications in the context of evolving regulatory considerations for cell- and tissue-based therapies. Techniques that minimize environmental exposure and intermediate handling steps may align with general principles aimed at preserving tissue integrity and reducing contamination risk. Although regulatory frameworks vary by region and continue to evolve, closed-circuit workflows may represent an area of interest for future investigation as standards for tissue processing and manipulation are further defined.

This is a technical report only and is limited by its descriptive nature, without formal comparative analysis or standardized quantitative outcome measures. Observations are based on clinical experience, and procedural variables were adjusted according to anatomic and clinical considerations rather than comparative cohorts, controls, or randomized evaluation. As such, no conclusions regarding clinical outcomes or comparative effectiveness can be drawn. No control group or comparative cohort was included. Further appropriately powered, controlled, randomized, blinded, prospective, comparative studies are needed to fully explore the potential merit of peristaltic pump systems.

### Future Research

Future studies should evaluate quantitative viability and cell function metrics, in vivo volumetric retention, and the potential impact of ASC/architecture preservation, along with the relationship between aspiration conditions (including pressure magnitude and duration) and adipose tissue integrity during harvest. Reporting graft quantity by mass (grams) rather than volume (cc or mL) may improve comparability across studies, as density differences related to fluid content, oil fraction, and processing techniques can introduce variability when volume alone is used. Additional work should include exploration of alternative methods for assessing cell viability and function beyond membrane integrity alone, including metabolic assays such as resazurin-to-resorufin reduction (Alamar Blue), alongside conventional live/dead assessments, with the goal of improving prediction of graft survival and biologic function, as well as correlation of technical parameters with clinical outcomes such as fat graft retention and contour stability. Objective assessment of graft retention using imaging modalities, such as photogrammetry or MRI, was not performed in this report but represents an important direction for future investigation.

## CONCLUSIONS

Peristaltic pump–assisted fat harvest/grafting represents a non–vacuum-based, semi-automated option to autologous fat transfer. This descriptive report demonstrates technical feasibility and consistent intraoperative handling characteristics. Peristaltic pump technique may represent an alternative approach for surgeons to consider. Further studies directly comparing this technique with traditional vacuum-based methods and fat viability assessments are needed to evaluate clinical outcomes.

## Supplemental Material

This article contains supplemental material located online at https://doi.org/10.1093/asjof/ojag122.

## Supplementary Material

ojag122_Supplementary_Data
